# Supplementary material for: The impact of Duration of Untreated Psychosis on functioning and quality of life over one year of Coordinated Specialty Care (CSC)
Source: PLoS One. 2025 Feb 10;20(2):e0312740. doi: 10.1371/journal.pone.0312740 (PMC11809850; doi:10.1371/journal.pone.0312740)
Supplement: S1 Table — (DOCX) [file pone.0312740.s001.docx]

Table 1s.

|  |  | 3 Month Cutoff | | | Median Cutoff | | |
| --- | --- | --- | --- | --- | --- | --- | --- |
| Outcome | Dup Total | Baseline | 6-month | 12-month | Baseline | 6-month (n =228) | 12-month |
| NLFET Status | High | 0.4 (0.33, 0.48) | 0.25 (0.18, 0.32) | 0.27 (0.2, 0.35) | 0.41 (0.32, 0.5) | 0.27 (0.19, 0.36) | 0.32 (0.23, 0.42) |
|  | Low | 0.33 (0.22, 0.47) | 0.09 (0.04, 0.22) | 0.09 (0.03, 0.23) | 0.36 (0.27, 0.46) | 0.14 (0.08, 0.23) | 0.12 (0.06, 0.21) |
|  | Odds Ratio | 1.32 (0.69, 2.51) | 3.25 (1.11, 9.54) | 3.59 (1.1, 11.73) | 1.24 (0.71, 2.16) | 2.25 (1.08, 4.71) | 3.4 (1.51, 7.68) |
|  | P-value | 0.3995 | 0.0318 | 0.0345 | 0.4501 | 0.031 | 0.0032 |
| GAF Current | High | 35.6 (27.6, 43.7) | 48.7 (40.6, 56.9) | 49.1 (40.9, 57.4) | 35.8 (27.7, 43.9) | 48.7 (40.5, 56.9) | 48.7 (40.3, 57.0) |
|  | Low | 33.1 (25.0, 41.2) | 57.4 (49.0, 65.8) | 54.6 (46.0, 63.3) | 34.5 (26.5, 42.5) | 54.2 (46.0, 62.4) | 53.2 (44.9, 61.5) |
|  | Delta |  | -11.2 (-15.7, -6.7) | 3.1 (-1.5, 7.8) |  | -6.8 (-11.0, -2.6) | 0.9 (-3.3, 5.1) |
|  | P-value |  | <.0001 | 0.1811 |  | 0.0016 | 0.6631 |
| GF: Role | High | 2.19 (0.58, 3.79) | 3.27 (1.67, 4.88) | 3.56 (1.94, 5.17) | 2.3 (0.7, 3.9) | 3.3 (1.6, 4.9) | 3.7 (2.0, 5.3) |
|  | Low | 2.18 (0.56, 3.80) | 4.19 (2.56, 5.83) | 3.86 (2.20, 5.51) | 2.2 (0.6, 3.8) | 4.0 (2.3, 5.6) | 3.8 (2.1, 5.4) |
|  | Delta |  | -0.93 (-1.60, -0.25) | 0.62 (-0.06, 1.29) |  | -0.8 (-1.4, -0.2) | 0.6 (-0.0, 1.2) |
|  | P-value |  | 0.0074 | 0.0728 |  | 0.0117 | 0.0522 |
| GF: Social | High | 4.09 (3.05, 5.14) | 4.76 (3.71, 5.80) | 4.69 (3.64, 5.74) | 4.2 (3.1, 5.3) | 4.8 (3.8, 5.9) | 4.8 (3.8, 5.9) |
|  | Low | 4.21 (3.15, 5.26) | 5.47 (4.41, 6.53) | 5.22 (4.15, 6.29) | 4.2 (3.2, 5.3) | 5.3 (4.2, 6.3) | 5.0 (4.0, 6.1) |
|  | Delta |  | -0.60 (-1.07, -0.13) | 0.18 (-0.29, 0.65) |  | -0.4 (-0.8, 0.0) | 0.2 (-0.2, 0.7) |
|  | P-value |  | 0.0124 | 0.4508 |  | 0.0585 | 0.2671 |
| Quality of Life | High | 59.5 (56.5, 62.6) | 70.5 (67.3, 73.7) | 69.9 (66.5, 73.4) | 58.6 (55.0, 62.2) | 68.6 (64.9, 72.4) | 68.4 (64.3, 72.4) |
|  | Low | 65.4 (60.4, 70.4) | 79.0 (73.6, 84.3) | 79.9 (74.2, 85.7) | 63.6 (59.8, 67.4) | 77.0 (73.0, 81.1) | 77.1 (72.7, 81.4) |
|  | Delta |  | -2.6 (-8.6, 3.5) | -1.5 (-7.6, 4.5) |  | -3.4 (-8.8, 2.1) | -0.3 (-5.8, 5.2) |
|  | P-value |  | 0.4037 | 0.617 |  | 0.2226 | 0.9231 |
| PANSS Positive | High |  | 7.9 (5.8, 9.9) | 7.7 (5.6, 9.8) |  | 8.6 (6.4, 10.8) | 8.5 (6.2, 10.8) |
|  | Low |  | 6.0 (3.8, 8.2) | 6.3 (4.0, 8.7) |  | 6.8 (4.8, 8.8) | 6.9 (4.8, 9.0) |
|  | Delta |  |  | 0.4 (-1.2, 2.1) |  |  | 0.2 (-1.4, 1.7) |
|  | P-value |  |  | 0.6078 |  |  | 0.8173 |
| PANSS Negative | High |  | 8.8 (6.8, 10.8) | 9.5 (7.5, 11.6) |  | 9.1 (7.0, 11.2) | 10.0 (7.8, 12.2) |
|  | Low |  | 8.7 (6.5, 10.9) | 9.2 (6.8, 11.5) |  | 8.6 (6.6, 10.6) | 9.0 (6.9, 11.1) |
|  | Delta |  |  | -0.2 (-2.2, 1.8) |  |  | -0.4 (-2.3, 1.4) |
|  | P-value |  |  | 0.8322 |  |  | 0.6362 |
| PANSS General | High |  | 15.4 (11.6, 19.3) | 15.0 (11.1, 18.9) |  | 15.4 (11.5, 19.4) | 15.3 (11.3, 19.3) |
|  | Low |  | 13.4 (9.3, 17.5) | 14.5 (10.4, 18.6) |  | 14.4 (10.6, 18.3) | 14.6 (10.7, 18.5) |
|  | Delta |  |  | 1.6 (-1.1, 4.2) |  |  | 0.4 (-2.0, 2.7) |
|  | P-value |  |  | 0.2406 |  |  | 0.7687 |
| PANSS Total | High |  | 33.0 (25.3, 40.8) | 33.2 (25.2, 41.1) |  | 34.0 (25.9, 42.1) | 34.6 (26.3, 42.9) |
|  | Low |  | 29.1 (21.1, 37.0) | 31.1 (22.8, 39.3) |  | 30.8 (23.2, 38.4) | 31.5 (23.8, 39.3) |
|  | Delta |  |  | 1.9 (-3.0, 6.7) |  |  | 0.2 (-4.3, 4.6) |
|  | P-value |  |  | 0.4504 |  |  | 0.9443 |

*Note. DUP: Duration of Untreated Psychosis; GAF: Global Assessment of Functioning; GF: Global Functioning; PANSS: Positive and Negative Syndrome Scale; QLS: Quality of Life Scale.*
