# Supplementary material for: The impact of Duration of Untreated Psychosis on functioning and quality of life over one year of Coordinated Specialty Care (CSC)
Source: PLoS One. 2025 Feb 10;20(2):e0312740. doi: 10.1371/journal.pone.0312740 (PMC11809850; doi:10.1371/journal.pone.0312740)
Supplement: S1 Fig — (DOCX) [file pone.0312740.s002.docx]

Figure 1s.
